# The Reproductive Toxicity of CdSe/ZnS Quantum Dots on the *in vivo* Ovarian Function and *in vitro* Fertilization

**DOI:** 10.1038/srep37677

**Published:** 2016-11-23

**Authors:** Gaixia Xu, Guimiao Lin, Suxia Lin, Na Wu, Yueyue Deng, Gang Feng, Qiang Chen, Junle Qu, Danni Chen, Siping Chen, Hanben Niu, Shujiang Mei, Ken-Tye Yong, Xiaomei Wang

**Affiliations:** 1 College of Optoelectronic Engineering, Key Laboratory of Optoelectronics Devices and Systems of Ministry of Education/Guangdong Province, Shenzhen University, Shenzhen 518060, P. R. China; 2 CINTRA CNRS/NTU/THALES, Singapore 637553, Singapore; 3 School of Medicine, The Research Institute of Urinary and Reproduction, The Engineering Lab of Synthetic Biology, Shenzhen Key laboratory of Biomedical Engineering, Shenzhen University, Shenzhen 518060, P. R. China; 4 Shenzhen Key Laboratory of Fertility Regulation, Department of Obstetrics and Gynecology, The University of Hong Kong-Shenzhen Hospital, Shenzhen 518053, China; 5 Shenzhen Center for Disease Control and Prevention, Shenzhen 518020, P. R. China; 6 School of Electrical and Electronic Engineering, Nanyang Technological University, Singapore 639798, Singapore

## Abstract

Despite the usefulness of quantum dots (QDs) in biomedicine and optoelectronics, their toxicity risks remain a major obstacle for clinical usages. Hence, we studied the reproductive toxicity of CdSe/ZnS QDs on two aspects, (i) *in vivo* ovarian functions and (ii) *in vitro* fertilization process. The body weight, estrous cycles, biodistribution of QDs, and oocyte maturation are evaluated on female mice treated with QDs. The mRNA level of the follicle-stimulating hormone receptor (FSHr) and luteinizing hormone receptor (LHr) in ovaries are assayed. Then, the matured cumulus-oocyte-complexes are harvested to co-culture with *in vitro* capacitated sperms, and the *in vitro* fertilization is performed. The result revealed that QDs are found in the ovaries, but no changes are detected on the behavior and estrous cycle on the female mice. The mRNA downregulations of FSHr and LHr are observed and the number of matured oocytes has shown a significant decrease when the QDs dosage was above 1.0 pmol/day. Additionally, we found the presence of QDs has reduced the *in vitro* fertilization success rate. This study highly suggests that the exposure of CdSe/ZnS QDs to female mice can cause adverse effects to the ovary functions and such QDs may have limited applications in clinical usage.

Quantum dots (QDs) have been extensively applied for biomedical applications such as cellular labeling, *in vivo* bioimaging, targeted drug delivery and disease diagnosis. These are mainly due to their excellent optical properties^1^,^2^,^3^,^4^. Besides biomedical applications, QDs are expected to be used increasingly in various optoelectronic devices, such as solar cells, sensors and light emitting diodes^5^. With the increasing applications of QDs worldwide, the potential toxicity concern of QDs towards the environment and living system remains a major debating topic to be discussed and addressed over the next few years. Previous studies have demonstrated that QDs can impair the cells and animals in many ways^6^. Generally, the size distribution, surface functions, morphology, dispersibility and aggregation state of QDs will induce different toxicity impacts. The cytotoxicity, pulmonary toxicity, neurotoxicity, nephrotoxicity and hepatotoxicity of QDs have been investigated and reported^7^,^8^,^9^,^10^,^11^,^12^,^13^,^14^,^15^. However, only several publications reported on the study of reproductive toxicity of QDs^16^,^17^.

In our previous study, we have co-cultured CdSe/CdS/ZnS QDs with immature oocytes or preantral follicles *in vitro* whereby the observation was on the invasion of QDs and the development of oocytes^18^,^19^. We found that the QDs were not able to transmigrate into the zona pellucida and thereby entered the oocyte. The QDs were found to be uptaken by the granulosa cells around the oocytes. The maturation rate of oocyte treated with QDs was found to decrease dramatically when compared to the control groups. Our result highlighted that the maturation of oocytes were significantly delayed in the presence of QDs. However, the cause of these abnormalities is not well understood and the toxicity mechanism of QDs in ovary is not known as well. It is also worth noting that this *in vitro* model cannot simulate the actual ovarian environment for regulating the maturation of oocytes. In this work, we established a sensitive but yet useful *in vivo* platform for studying and analyzing the potential reproductive toxicity of QDs on the ovarian function and *in vitro* fertilization. This model simulates an *in vivo* ovarian environment and will be a valuable platform for nanoparticle reproductive toxicity evaluation. This is especially true for those nanoparticles that will be used for *in vivo* imaging or therapy.

In this work, the 6-week-old BABL/c female mice have been subcutaneously injected with CdSe/ZnS QDs for 14 days, at dosage of 0.1 pmol, 1.0 pmol and 5.0 pmol per day per mouse, and the effects of QDs on the oogenesis and the ovarian functions were investigated *in vivo*. The matured oocytes in each group were harvested and co-cultured with capacitated sperms to evaluate the *in vitro* fertilization potential. Our result showed that some QDs were found to be accumulated in the mice’s ovaries. We have discovered that the mRNA levels of LHr and FSHr in ovarian tissue were greatly reduced when the injected QDs dosage was above 1.0 pmol leading to a delay in the mice’s oocyte maturation. In addition, the QDs exposure on the female mice has disrupted the fertilization activity of matured oocytes. However, the overall morphology of oocytes remained to be normal. Our study offers a significant and valuable platform for future research on the potential reproductive toxicity of various QDs *in vivo* and *in vitro* and thereby providing useful information in guiding the QDs community to engineer safer QDs for specific biomedical applications.

## Results and Discussion

### Characterization of the CdSe/ZnS QDs

**Figure 1:**
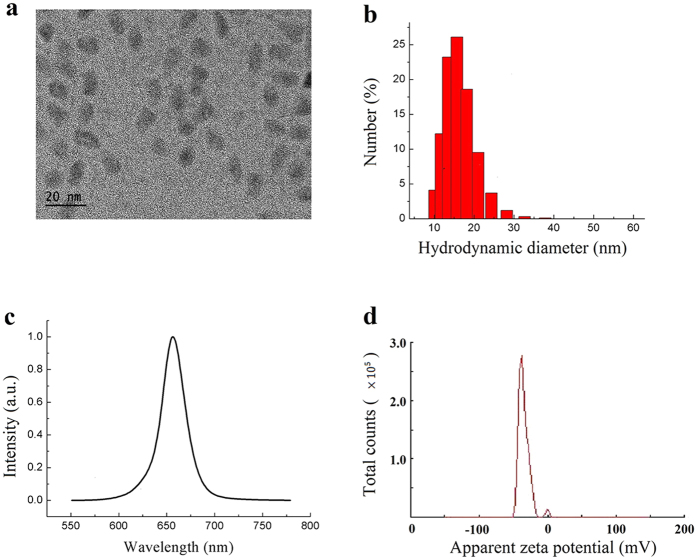
The characterization of CdSe/ZnS QDs. (**a**) The TEM image of QDs, (**b**) The hydrodynamic size distribution of QDs, (**c**) The fluorescence emission of QDs, (**d**) The Zeta potential.

Before the biological experiments were performed, the CdSe/ZnS QDs formulation was characterized. The TEM image showed that the diameter of the CdSe/ZnS core/shell QDs was 9.79 ± 2.185 nm (Fig. 1a). The CdSe/ZnS QDs were carboxylate functionalized, which was then used for experimental purpose. The carboxylated QDs was further characterized using dynamic light scattering (DLS) technique. The hydrodynamic diameter of the particles was determined to be 14.55 ± 4.157 nm, and the polydispersity index was 0.286 (Fig. 1b). The fluorescence emission peak was centered at 655 nm (Fig. 1c), and the zeta potential was measured to be −35.1 mV (Fig. 1d). The prepared QDs formulation was kept at 4 °C and no aggregation or precipitation was observed for more than 3 months.

### Effects of QDs on the body weight and organ index

**Figure 2:**
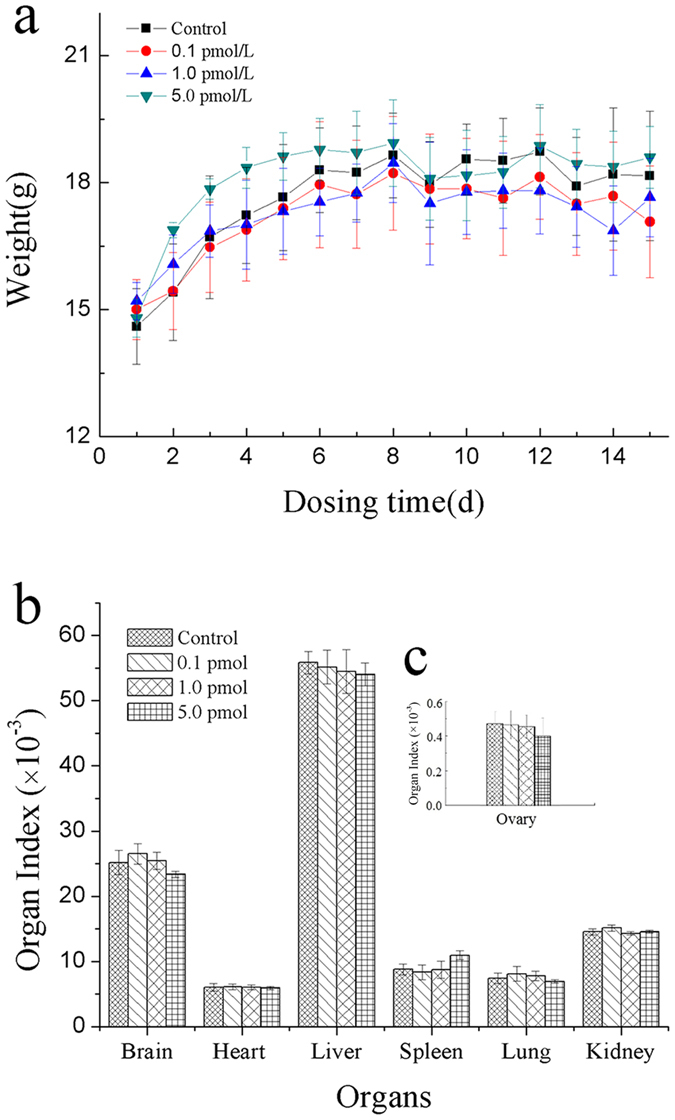
Effects of QDs on mouse body weight and organ index. The data was presented as the mean ± SD. (**a**) The body weigth of mice in various groups. n = 6. (**b**) The general organ index after treatment with QDs for 14 days. n = 4. (**c**) The ovary organ index after treatment with QDs for 14 days. n = 4.

The body weights of all the mice were recorded every day before and after QDs treatment. No changes in eating, drinking, fur color, and exploratory behavior has been observed for the mice treated with QDs. The body weight plot showed that the weights of the mice increased normally and no difference was observed between control and QDs-treated groups (p > 0.05; Fig. 2a). This suggested that the QDs (≤5.0 pmol/day/mouse consecutively for 14 days) did not cause any adverse effect to the body weight of the mice. After 14 days of administrations, the mice were sacrificed and the major organs and ovaries were harvested. The organ indexes (organ weight/body weight) of heart, liver, spleen, lung, kidney and brain were calculated and compared (Fig. 2b). The result showed that the QDs had no obvious effect on the organ indexes, which was consistent with our Immunotoxicity assessment study of CdSe/ZnS on BABL/c mice^20^. Since we wanted to study the reproductive toxicity of QDs for female mice, the organ index of ovary was recorded as well but no obvious difference was detected between the control and treated groups (Fig. 2c).

### Biodistribution of QDs in organs

**Figure 3:**
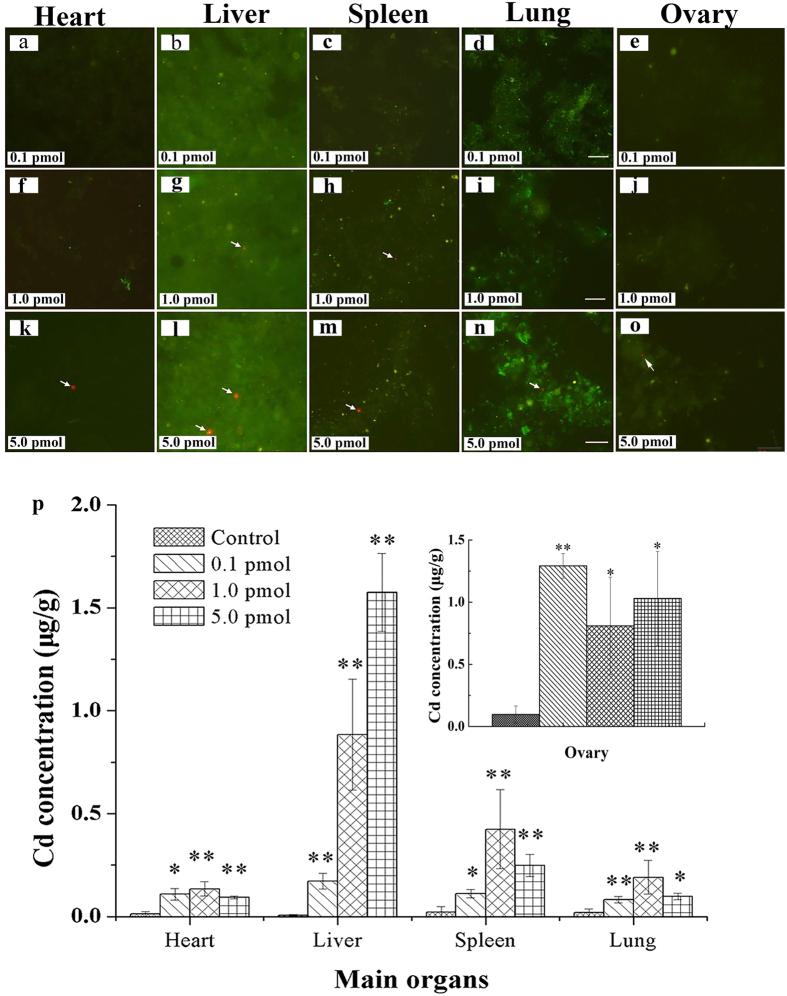
Biodistribution of QDs in main organs after 14 days QDs treatment. (**a–o**) The typical fluorescence images of QDs distributed in organs, n = 7. Arrows pointed to CdSe/ZnS QDs with red fluorescence, Scale bar: 20 μm. (**p**) The ICP-MS analysis of Cd concentrations in organs. The data was presented as the mean ± SD, n = 4.

Considering the biopotency and heterogeneous structures of various organs, the QDs might be undetectable in thin frozen slides, which would lead to false conclusion. Thus, we harvested the main organs, including heart, liver, spleen, and lung, with size of 3 × 3 × 3 mm^3^, and ground the tissues with a homogenizer. Then, the smeared tissues on the glass slides were imaged and analyzed by a fluorescence microscope. The results showed that QDs fluorescence signals were detected in all the organs treated with 5.0 pmol QDs (Fig. 3k~3o). As for the organs obtained from the mice treated with 1.0 pmol QDs, less QDs were found in spleen and liver, and no QDs fluorescence signal was detected in the heart, lung and ovary (Fig. 3f~3j). There was no QDs fluorescence signal detected for the major organs removed from the mice treated with 0.1 pmol QDs. In addition, we performed a detail inductive coupled plasma mass spectrometry (ICP-MS) analysis on the harvested organs. Our result revealed that, for 5 pmol/mouse of QDs, the concentrations of Cd, Se and Zn corresponded to 0.78 mg/kg, 0.023 mg/kg, and 0.0691 mg/kg, respectively. The concentration of Cd in each of the major organs was measured and analyzed (Fig. 3p). This result confirmed that the QDs were accumulated in the major organs.

It has been well documented that the threshold particle size for renal clearance is around 5.5 nm^21^. Thus, the QDs with the size greater than 5.5 nm will accumulate mainly in the liver, spleen and kidney. This will increase the potential risk for QDs to be degraded in the body and subsequently causing *in vivo* toxicity. Furthermore, this is particularly true for Cd-based QDs where the risk increases dramatically since the clearance of cadmium from the body is very slow, e.g. 20 years^22^,^23^. The accumulation of QDs were mainly found in reticuloendothelial system (RES) organs, such as liver and spleen^20^,^24^.

In our previous work, CdSe/CdS/ZnS QDs at dosage of 28.9 pmol/mouse was injected into female mice, and the distribution of QDs in ovary was analyzed^25^. Notably, the CdSe/CdS/ZnS QDs were found in ovary up to 14 days after administration. Ovary is the organ for production and periodical release of the female gametes, and secretes estrogen, testosterone and progesterone to regulate the estrous cycle and ovulation. Since the ovary and oocytes are highly sensitive towards their microenvironment, the presence of QDs in ovary might induce some adverse effects on oocyte and fertility^26^.

In this work, the distribution of QDs in ovary was determined. The morphology of ovary was observed under stereomicroscope and no obvious morphological change was found in treated groups when compared to the control group. The fluorescence images were shown in Fig. 3e,j and o. The green fluorescence signal originated from the auto fluorescence of the biological tissues and the red fluorescence signal was generated from QD particles. When the mice were treated with QDs at a lower dose (≤1.0 pmol), there was no red fluorescence signal (Fig. 3e and j) and this was similar to the control group treated with PBS buffer. When the mouse was treated at a higher dose (5.0 pmol/mouse), the red fluorescence signal was detected in the ovary (Fig. 3o). The data suggested that subcutaneous injection of QDs into the female mice would lead to the accumulation of QDs in the ovary. The ICP-MS analysis confirmed the accumulation of QDs in ovary (Fig. 3p).

In our previous work, we demonstrated that most of the QDs would accumulate in RES organs when tail vein injection was used^20^. This was because the QDs were recognized and removed from the blood stream by the RES organs. However, when the QDs were introduced to the body through subcutaneous injection, the accumulation and the toxicity level of the particles will be different in comparison to tail vein injection. Our fluorescence imaging experiments showed that some fractions of QDs were found in the ovary. This indicated that subcutaneous injection might affect the overall function of the ovary. The ICP-MS result indicated that the injected QDs were distributed in RES organs and ovary. More importantly, the total concentration of Cd in ovary was found to be as high as those in the liver and spleen. This trend was different from that of using tail vein injection method. We deduce that the accumulation of Cd in ovary may originate from two factors, which are local uptake and blood circulation. When the QDs were injected into the mouse body, some fractions of carboxylated nanocrystals will be aggregated in the presence of biomolecules from the local environment and arrested in the location near to the injection point, such as ovary. Subsequently they will be taken up by the ovary through diffusion. The rest of the nanoparticles will enter the blood stream.

Upon comparing to our other reproductive toxicity of QDs^25^, there were two main differences. First, in this work, the cumulative dosage of CdSe/ZnS QDs locally injected were 70 pmol/mouse, 14 pmol/mouse and 1.4 pmol/mouse, respectively. The QDs fluorescence signals were only found in the highest-dosage treated group which was 2.4 times more than the dosage used in our previous work (28.9 pmol/mouse). Second, our previous study employed tail vein injection approach and the distribution of the nanocrystals were analyzed after 14 days. The present work employed subcutaneous injection approach and the injection was carried out daily for 14 days consecutively. Similarly, QD signals could also be found in ovarian tissue. The result indicated that no matter which injection method was used, the accumulation and retention of QDs in ovary will take place after 14 days of treatment.

### Effect of QDs on mRNA level of LHr and FSHr

**Figure 4:**
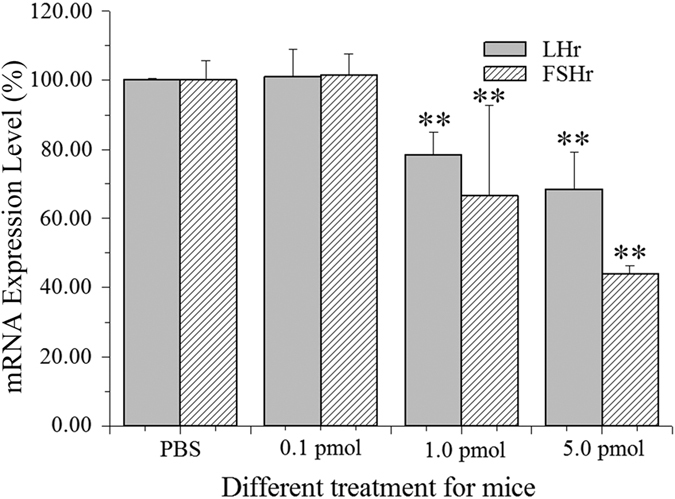
The mRNA expression levels of LHr and FSHr in ovaries of different groups. The data was presented as the percentage mean ± SD. n = 7.

The amounts of FSHr and LHr are often used to evaluate the ovary function^27^. In this work, the effects of QDs on mRNA level of LHr and FSHr in ovary were studied. The real-time PCR (RT-PCR) was performed to compare the mRNA expression levels of FSHr and LHr in ovarian tissue. The statistical data was shown in Fig. 4. The relative gene expression levels of LHr and FSHr in control group were 100 ± 0.291% and 100 ± 5.321%, respectively. When 0.1 pmol QDs was used, the relative gene expression levels of LHr and FSHr were 100.31 ± 8.092% and 101.42 ± 6.231%, respectively. These data showed no significant difference between 0.1 pmol group and control group (p > 0.05). If the concentration of QDs was increased to 1.0 pmol, the gene expression levels of LHr and FSHr were reduced to 78.21 ± 10.13% and 66.64 ± 26.39%, which showed a significant change when compared to the untreated ones (p < 0.01). However, when the QDs concentration was increased to 5.0 pmol, the gene expression levels of LHr and FSHr decreased drastically to 68.49 ± 10.27% and 44.00 ± 2.84%. It was obvious that the mRNA levels of LHr and FSHr in ovary were proportionally affected by the overall doses of QDs treated to the animals (concentration ≥ 1.0 pmol). This has shown that the exposure of QDs *in vivo* has greatly downregulated the mRNA expressions of LHr and FSHr.

In the ovary, the aromatase activity and estradiol production within granulosa cells are stimulated by FSH and mainly dependent on FSH receptor density^28^. The LHr specifically acts to regulate the enzyme cholesterol side chain cleaving enzyme, which affects the conversion of cholesterol into androgen precursors, resulting in the amount of steroid hormones productions change^29^. Thus, we hypothesize that the mRNA downregulations of FSHr and LHr could decrease the steroid production in ovary. Stelzer *et al.* demonstrated that the accumulation of gold nanoparticles in granulosa cells had disturbed the steroid biosynthesis and reduced the estradiol production^30^. They reported that the nanoparticles perturbed the mitochondrial membranes of granulosa cells, and eventually affected the steroidogenesis via an oxidative stress-mediated mechanism. Our previous work also confirmed that the QDs could be uptaken by granulosa cells and theca cells *in vitro*^19^,^31^.

Taken together, we considered that there were two possible pathways for QDs to affect the mRNA expression levels of LHr and FSHr. In the first scenario, the QDs were uptaken by granulose cells and thecal cells and subsequently led to lysosomal enlargement and mitochondrial swelling and rounding up^32^, which indicated that, the cells underwent oxidative stress. It is known that more than 90% of the energy used in mammalian cells is provided by the mitochondria through oxidative phosphorylation. Thus, the impairment of mitochondrial inevitably decreased the protein synthesis and expression, such as LH and FSH receptors. In the second scenario, the prolonged retention of QDs in mice induced the release of Cd^2+^ and thereby altering the membrane integrity of ovarian granulose cells and theca cells^32^. The development of follicles and the proliferation of granulosa cells were inhibited. As a result, the expression of the receptors on cellular membrane was decreased.

### Effect of QDs on mouse estrous cycle

The estrous cycle is a distinctive feature of most female mammalians, which comprises the periodic physiologic changes that are induced by hormones. In this study, a careful observation of mouse estrous cycle was performed by imaging the vaginal secretion (Figure S1). In diestrus, the vaginal secretions were generally spherical leukocytes (mostly neutrophils), which gathered piles with dark stained horseshoe-shaped nucleus. During proestrus, the vaginal secretions included dispersal nucleated epithelial cells. As for estrus, there were cornified epithelial cells with the polygon shapes, whose nucleus shrank and even disappeared, leaving “empty cells”. During metaestrus, there were a few cornified epithelial cells and nucleated epithelial cells. The leukocytes were also observed. The images of the vaginal cytology showed that there was no morphology change between experimental and control groups in the four estrous phases. Additionally, the exploratory behaviors of all the groups had no obvious difference. These results qualitatively suggested that the QDs had no impact on the vaginal smear cytology.

In order to evaluate quantitatively the effect of QDs on estrous cycle during the experiment, the serum samples were collected. Then, the hormones, estrogen and progesterone, were assayed for the last 8 days consecutively. Generally, the mouse estrous cycle was estimated to be around 5 days. In each estrous cycle, the concentrations of estrogen and progesterone in the serum were measured by identifying their corresponding peaks in the plot. In this case, the measurement covered for at least one complete estrous cycle^33^. The peak value for every single day was used as the starting point to graph the concentration trends of estrogen and progesterone. Our result showed that there was no significant difference between control and treated groups. The patterns of estrogen and progesterone in one complete estrous cycle obtained in this work were in good agreement with other published works^34^.

Both of the results as seen above suggested that the applied QDs concentration at ≤5.0 pmol had negligible effect on the female mice estrous cycle and this implied that the QDs did not affect the hormone releases or have any changes that could lead towards a negative impact to the estrous cycle.

Generally, the estrous cycle is regulated by a complex and systematic reproductive system in female mammals. It includes the regulatory hypothalamic system that releases hormone in pulses, the pituitary that secretes FSH and LH, and the ovary that produces estrogens and progesterone. Our work showed that the decrease of mRNA expression levels of hormone receptors in ovary was unable to affect the estrous cycle within our examination period.

### Effect of QDs on oocyte nuclear maturation

**Figure 5:**
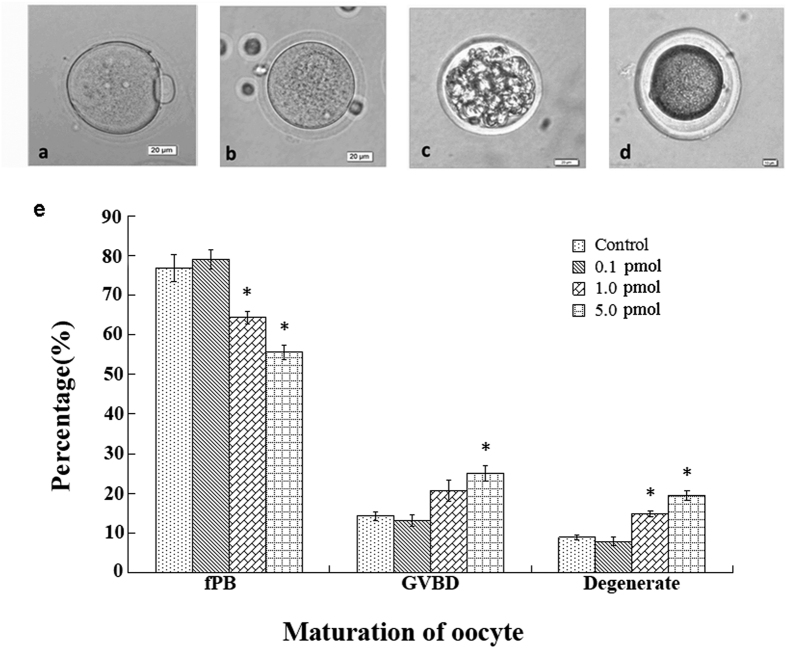
The effect of QDs on oocyte maturation. (**a–d**) Typical oocytes obtained from the mouse oviduct ampulla treated with 5.0 pmol QDs. (**a**) matured oocyte with fPB, (**b**) GVBD oocyte, (**c,d**) degenerated oocyte. Scale bar: 20 μm. (**e**) The statistic data of oocyte maturation. The data was presented as the percentage mean ± SD. (control, n = 112; 0.1 pmol, n = 114; 1.0 pmol, n = 101; 5.0 pmol, n = 108).

It was reported that the dysfunction of granulosa cells would alter the steroidogenesis and hormone production and thereby affecting the oocyte development^30^. In order to estimate the effects of QDs on the oogenesis, the female mice were superovulated with hormones of PMSG and hCG. Subsequently the oocytes were obtained and analyzed. Figure 5 showed the typical morphology of oocytes at different maturation stages. The normal nucleus-matured oocyte with first polar body (fPB), was shown in Fig. 5a. The germinal vesicle breakdown (GVBD) oocyte was shown in Fig. 5b. In such immature oocyte, the membrane of germinal vesicle broke and the first polar body was not released. Lastly, the parthenogenetic oocytes and the nucleoplasm shrunken oocytes were classified as degenerated oocytes (Fig. 5c and d). The statistical analysis on the maturation of superovulated oocytes was performed (Fig. 5e). In comparison to the control group, the rate of matured oocytes with fPB at higher QDs dosage (≥1.0 pmol/day/mouse) was found to decrease (p < 0.05). Correspondingly, the rate of immature GVBD oocytes and the degenerated oocytes increased dramatically (p < 0.05).

As discussed previously, the presence of QDs has caused the mRNA expression levels of FSHr and LHr to decrease. It was known that the FSH, LH and estrogen play important roles for the development of follicles and oocyte. FSH binds with FSHr on the granulosa cells which accelerates the granulosa cell proliferation. Then, it stimulates the aromatase activity, converts the androgens to estrogens, and induces the formation of LH receptor on the theca cells. LH binds with LHr on the theca cells which accelerates the theca cell proliferation. Then, it stimulates theca cells to produce androgens which will be transported into granulosa cell and finally converted to estrogens. The FSH, LH and estrogen are playing active roles in maturing the oocytes. During the regulation, the estrogen is the key factor. Due to the decrease of FSHr and LHr, this will inevitably lead to the lower production of estrogen, and therefore the maturation rate of oocytes will decrease for QDs treated samples. Additionally, our result also demonstrated that the QDs inhibited the releasing of the first polar body and delayed the oocyte nuclear maturation.

### Effect of QDs on *in vitro* fertilization

**Figure 6:**
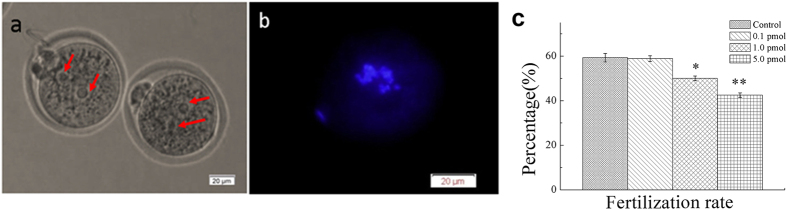
The effect of QDs for *in vitro* fertilization. (**a**) The typical microscopic images of the zygotes. Arrows pointed to the regular male and female pronuclei; (**b**) The fluorescence image of DAPI- stained chromosomes. Scale bar: 20 μm. (**c**) The statistic data of *in vitro* fertilization. The data was presented as the percentage mean ± SD. (control, n = 86; 0.1 pmol, n = 90, 1.0 pmol, n = 76; 5.0 pmol, n = 73).

To further investigate the impacts of QDs on the fertilization of the mice treated with QDs for 14 days, the cumulus-oocyte-complexes (COCs) containing the matured fPB oocyte were harvested and co-cultured with capacitated sperms. All the co-cultured oocytes and sperms were fixed and stained by DAPI. The two-cell zygotes were observed under microscope. In Fig. 6a, there was a residual sperm tail near the left oocyte. In addition, there were obvious regular male and female pronuclei in each oocyte. The fluorescence image showed the two sets of chromosomes inside one oocyte, which confirmed the success of *in vitro* fertilization (Fig. 6b). The blue fluorescence from chromosome near the zona pellucida was originated from the first polar body. The number of zygote was counted in all groups. The statistic fertilization rates were shown in Fig. 6c. For control group, the fertilization rate was determined to be (59.30 ± 1.91)%, which had negligible differences with that of the group treated with 0.1 pmol QDs ((58.89 ± 1.30) %, P > 0.05). When the mice were treated with 1.0 pmol and 5.0 pmol QDs, the fertilization rate decreased to (50.00 ± 1.07) % and (42.50 ± 1.03) %, respectively. These results suggested that the higher dosage of QDs might reduce the fertilization potential of matured oocyte.

Considering the complexity of oocyte maturation, we proposed three possible mechanisms that will induce the weakening of *in vitro* fertilization process. In the first case, the oocyte with immatured cytoplasm contained in COCs will decrease the *in vitro* fertilization. The oocyte maturation includes nuclear maturation and cytoplasmic maturation^19^. The oocyte nucleus maturation characterizes with a released first polar body. The oocyte cytoplasmic maturation mainly involves reorganization of the cytoskeletal filaments and redistribution of cytoplasmic organelles, including the mitochondria, cortical granules and endoplasmic reticulum. Since the oocyte maturation was FSH and LH dependent, the decline of FSHr and LHr would greatly affect the oocyte cytoplasmic maturation. Because the oocytes were surrounded by cumulus cells, thus it is difficult to clearly check the oocyte cytoplasm. In this case, there might be some COCs with cytoplasmic immature oocytes that were also harvested for *in vitro* fertilization, which inevitably induced the lower success of *in vitro* fertilization.

In the second scenario, the oocyte at the early stage of the nuclear maturation decreases the *in vitro* fertilization. The subtle differences of first polar body, such as size, smoothness and integrity will reflect the nuclear maturation stage of the oocyte. In the *in vitro* fertilization experiment, the COCs, but not the solitary oocyte, were taken out, observed, selected, and cultured with sperms. Because the oocyte was encapsulated by the cumulus cells, the details of first polar body could not be identified clearly. Thus, some oocytes at the early stage of nuclear maturation might be introduced into the *in vitro* culture system, and finally reduced the success rate of *in vitro* fertilization.

In the third scenario, the effect of Cd^2+^ on COCs decreased the *in vitro* fertilization. The retention of QDs in biological environment will induce the Cd^2+^ releasing^35^,^36^,^37^ Thompson *et al.* reviewed that the Cd suppressed the cumulus expansion and reduced oocyte maturation^38^. In this study, The ICP-MS discovered that the concentration of Cd in ovary was as high as that in liver and spleen (Fig. 3o). However, the fluorescence of QDs in ovary was less than that in RES organs, which indicated that majority of the QDs were uptaken by RES organs and some of the nanocrystals may underwent partial degradation^24^. That was inevitable to affect the COCs development, and then decreased the *in vitro* fertilization.

For reproductive toxicity study on QDs, the preantral follicle *in vitro* growth system had been demonstrated to be 10 times more sensitive than the conventional *in vivo* animals for reproductive medical and biological investigations^19^,^39^, the lack of *in vivo* environment and the coordination of pituitary, ovary and hypothalamus will lead to inconclusive judgement, especially for hormones-relevant studies. The *in vivo* mammals provided an actual ovarian environment for regulating the maturation of oocytes^20^,^40^. However, they were more expensive and time-consuming. Chan *et al.* developed a platform for QDs reproductive toxicity by exposing oocytes to QDs *in vitro*, and then performing the *in vitro*/*in vivo* assays, including oocyte maturation, fertilization, and resulting pre-implantation and post-implantation development of blastocysts^17^. This strategy shortened the experimental period and reduced the consumption of animals. Nevertheless, the implantation of embryos increased the difficulty of the experiment, and the results greatly depended on the operator’s skills.

In the present work, the female mice were treated with QDs *in vivo*, and the corresponding effects caused by QDs were assessed, including the QDs biodistribution, the mRNA expression levels of hormone receptors, the oocyte nuclear maturation, the estrous cycle and the *in vitro* fertilization. This model revealed the true responses from the living animals exposed with exogenous nanoparticles. Also, this platform shortened the experiment period and significantly decreased the experimental cost. Such platform will be a useful tool for evaluating the *in vivo* reproductive toxicity of nanoparticles.

## Conclusion

Many reports have shown that some nanoparticle formulations might cause adverse effects *in vivo* because of the small particles size and instability of the nanoformulations^41^. It is important to understand the QDs toxicity *in vitro* and *in vivo* before they could be ultilized widely in biomedical research. To our knowledge, this was the first time to report the effect of CdSe/ZnS QDs for the *in vivo* ovarian function and the subsequent effect on the fertilization *in vitro*. The results showed that the QDs disturbed the oocyte maturation, reduced the mRNA expression levels of hormone receptors, and decreased the *in vitro* fertilization potential. Hence, it is necessary to take into account the safe dose of QDs for the reproductive system when we apply the QDs as the fluorescent probes for reproductive medicine. This study also provided a simple and practicable platform to investigate the reproductive toxicity of Cd-based QDs, which will be helpful for understanding the mechanism of interaction between animals and the engineered nanoparticles.

## Methods

### Chemicals

Pregnant mare serum gonadotrophin (PMSG), human chorionic gonadotrophin (hCG), hyaluronidase, N-2-Hydroxyethylpiperazine-N′−2- ethanesulfonic acid (HEPES), isolation medium M2 (containing 200 IU·mL^−1^ penicillin + 200 IU·mL^−1^ streptomycin + 20 mmol·L^−1^ HEPES, supplemented with 5.0% FBS), and DAPI were purchased from the Sigma. Penicillin and streptomycin were purchased from North China Pharmaceutical Group Corporation. The other chemicals were purchased from Sinopharm Chemical Reagent Co. Ltd. The CdSe/ZnS core/shell QDs (Qdot^®^ 655 ITK™ carboxyl quantum dots, Catalog nos.Q21321MP) used in this work were purchased from Invitrogen. All chemicals were used as received. The other chemicals were of reagent grade and were used without further purification.

### Animal

The 6-week-old SPF grade female and male BABL/c mice were purchased from the Experimental Animal Center of Guangdong Province [SCXK (Guangdong) 2008–0002], housed in groups in a temperature and light-controlled room at 23–25 °C, on a 12-hour light and 12-hour darkness rotating cycle, and fed with pellet food and water as desired. All the mice have adapted to the new environment for one week. 28 tested female mice were divided into four groups randomly. The 3 experimental groups were treated with 100 μL QDs at the dosage of 5.0, 1.0 and 0.1 pmol/day/mouse, respectively, for 14 consecutive days by subcutaneous injection. The control group was injected with 100 μL/day PBS for 14 consecutive days, too. Animal handling was carried out in accordance with the standard animal husbandry practice and regulation of the Laboratory Animals Center of Shenzhen University. All the animal experiments and maintenance were approved by the Laboratory Animal Ethics Committee of Shenzhen University.

### The estrous cycle identification and hormone detection

The changes of estrous cycle of the four groups had been observed and recorded from the mice administration one week ago till the mice were harvested. Every morning (8:00 am~9:00 am), the mouse was fastened on the table and then the vaginal opening was exposed before the vulva was preliminary checked. Then, a small amount of sterile saline was dropped on the vaginal opening. The tail and waist of the mouse were slightly relaxed, and the saline was sucked into vagina. When we gently pressed the mouse’s waist, the vaginal secretion with saline flowed out. The smear was dried and fixed with 95% alcohol for 5 mins. The eosin dye was used to stain the samples for 15 minutes. Finally, the slides were washed thrice and dried before microscopic observation.

The levels of progesterone and estradiol in serum were measured using enzyme-linked immunosorbent assay (ELISA) (Cloud-Clone Corp., USA), according to the manufacturer’s instructions.

### Biodistribution analysis

After finishing the administration, all the organs of different groups were collected with the size of 3 × 3 × 3 mm^3^, washed by physiological saline, ground with a homogenizer and diluted with 200 μL phosphate buffer solutions (PBS). Then, the smear slides were prepared and observed by fluorescence microscope ((BX51, Olympus, Japan).

After the mice were sacrificed, the major organs (heart, liver, spleen, lung and ovary) were collected from animals. Before ICP-MS analysis, the tissues were cut into pieces, digested with 6 mL 65% HNO_3_ and 2 mL 30% H_2_O_2_ at 200 °C for 30 minutes by a microwave acid digestion apparatus (ETHOS ONE, Milestone, Italy). Then the solution was diluted 10 times with deionized water and analyzed using an ICP-MS machine (7500C1, Agilent, USA).The elemental concentrations of cadmium was calculated according to the standard protocol^20^, and the minimum detection limit was 0.05 μg/L.

### Total RNA extraction from ovarian tissue and real-time quantitative PCR

The total RNA from mice ovary was extracted using Trizol method (Invitrogen) and measured by a spectrophotometer (Nano-Drop ND-1000). The total RNA (2 μg) was reverse transcribed to cDNA using the reverse transcriptase kit (Promega) according to the manufacturer’s instructions. Then Real-time quantitative PCR analysis was performed using SYBR Green kit (Progema) at the temperature conditions shown in Table S1 and S2. Relative mRNA expression levels of LHr and FSHr were quantified by the threshold cycle number (CT) and controlled for quantity of RNA input by performing measurements on (GAPDH). The data are expressed as transcript accumulation index (TAI = 2^−△△CT^) as following,


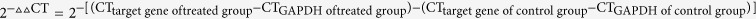


The mRNA expression levels of LHr and FSHr in treated group were normalized to those in group control, assigning the mRNA expression levels in group control as 100%.

The structure diagram of primer design was shown in Figure S2, the oligonucleotide primers were synthesized by BGI-Shenzhen (China) and sequences were as following^42^,^43^,

1. FSHr/F:5′-TGTCATCACTGGCTGTGTCAT-3′,FSHr/R:5′-GATGTACAGCAGATTGTTAGCC-3′.
2. LHr/F:5′-AAGCAGTCACAGCTGCACTCT-3′,LHr/R:5′-TTCAGACAGATTGAGGAGGTTG-3′.
3. GAPDH/F:5′- AACGACCCCTTCATTGACC-3′, GAPDH/R:5′- TCCACGACATACTCAGCACC-3′.

The amplification plots and melting plots were shown as Figure S3, which were smooth and without stray peak. The agarose gel electrophoresis of quantitative PCR product was performed and the results were shown in Figure S4, which confirmed the molecular weight of LHr and FSHr. The Figure S3 and S4 demonstrated that the design of the experimental primer was successful and specific, and the quantitative results were valid.

### Superovulation and collection of matured oocytes

Generally, it takes 14 days for maturation of oocyte from preantral follicle to oocyte with first polar body. Thus, we collected oocytes and organs at the endpoints of 14 days after QDs treatment. During the diestrus, before the endpoint of administration with PBS or QDs, the female mice were induced to superovulate oocytes by an injection of 10 IU PMSG followed by an injection of 10 IU hCG 48 hours later. After 18 to 20 hours later, the mice were sacrificed by cervical dislocation for the experiments. The normal mice ovaries are anatomically next to fallopian tubes. The matured follicles locate in the ampulla of fallopian tubes. The matured oocytes, which have recovered and finished the first meiotic division, are released directly from the follicles into the oviduct ampulla magnum. During our experiment, the oviduct ampullar magnum of mice were harvested and punctured with needles. Then, the cumulus-oocyte-complexes (COCs) were released. After being transferred into a culture dish, the structure of a single COC was observed clearly, whereby oocyte was at the center, surrounded by multi-layers of cumulus cells.

### Sperm capacitation

Before being killed, the male BABL/c mice were mated once every 4 days at interval, and had been abstinent for 4 to 6 days. One hour before the matured oocytes were obtained, the male mice were killed. The epididymis and vas deferens were harvested and transferred into the pre-warmed human tubal fluid (HTF) culture medium at 37 °C. The excess fat tissue was removed by needles under the stereoscopy. Then, the epididymis and vas deferens were washed twice and cultured in fresh HTF culture medium. The cauda epididymis was punctured by needles and the sperms were released. The sperms were transferred into pre-warmed HTF culture medium (37 °C, 5% CO_2_) and capacitated for 30 to 45 minutes.

### *In vitro* fertilization

The typical microscopic images of epididymis and sperm stained by H&E were shown as Figure S5a and b. The oviduct ampulla magnum and the COCs were shown in Figure S5c and d. The COCs were transferred into the balanced fertilization medium and washed twice while the sperms were capacitated. The oocyte pipette was used to blow the COCs to disperse the granulosa cells around the oocyte. Then, ten COCs were transferred into the one drop of HTF culture medium (40 μL). The capacitated sperms were also added into the same drop, with final density of 0.5~1 × 10^6^ mL^−1^. The culture dishes were put into humid incubator (37 °C, 5% CO_2_) for *in vitro* fertilization for 5~8 hrs. All the procedures were operated quickly and under reduced light exposure. Once the capacitated sperms were added into the HTF culture medium, the sperms immediately surrounded the COCs. The sperms secreted hyaluronidase to digest the cumulus cells and arrived at the zona pellucida of oocytes. Then, the fertilization process started.

### Statistics

The data were analyzed by one-way Anova followed by Dunnet’s t-test, presented as the percentage or mean ± standard deviation (SD), and considered statistically significant at *P < 0.05, **P < 0.01. All statistical calculations were done with the SPSS software package.

## Additional Information

**How to cite this article**: Xu, G. *et al.* The Reproductive Toxicity of CdSe/ZnS Quantum Dots on the *in vivo* Ovarian Function and *in vitro* Fertilization. *Sci. Rep.* **6**, 37677; doi: 10.1038/srep37677 (2016).

**Publisher's note:** Springer Nature remains neutral with regard to jurisdictional claims in published maps and institutional affiliations.

## Supplementary Material

Supplementary Information
