# Supplementary material for: The Reproductive Toxicity of CdSe/ZnS Quantum Dots on the in vivo Ovarian Function and in vitro Fertilization
Source: Sci Rep. 2016 Nov 23;6:37677. doi: 10.1038/srep37677 (PMC5120285; doi:10.1038/srep37677)
Supplement: Supplementary Information [file srep37677-s1.pdf]

Supplementary Information

The reproductive toxicity of CdSe/ZnS quantum dots on the *in vivo* ovarian function and *in vitro* fertilization

Gaixia Xu, Guimiao Lin, Suxia Lin, Na Wu, Yueyue Deng, Gang Feng, Qiang Chen, Junle Qu, Danni Chen, Siping Chen, Hanben Niu, Shujiang Mei, Ken-Tye Yong and Xiaomei Wang

Results

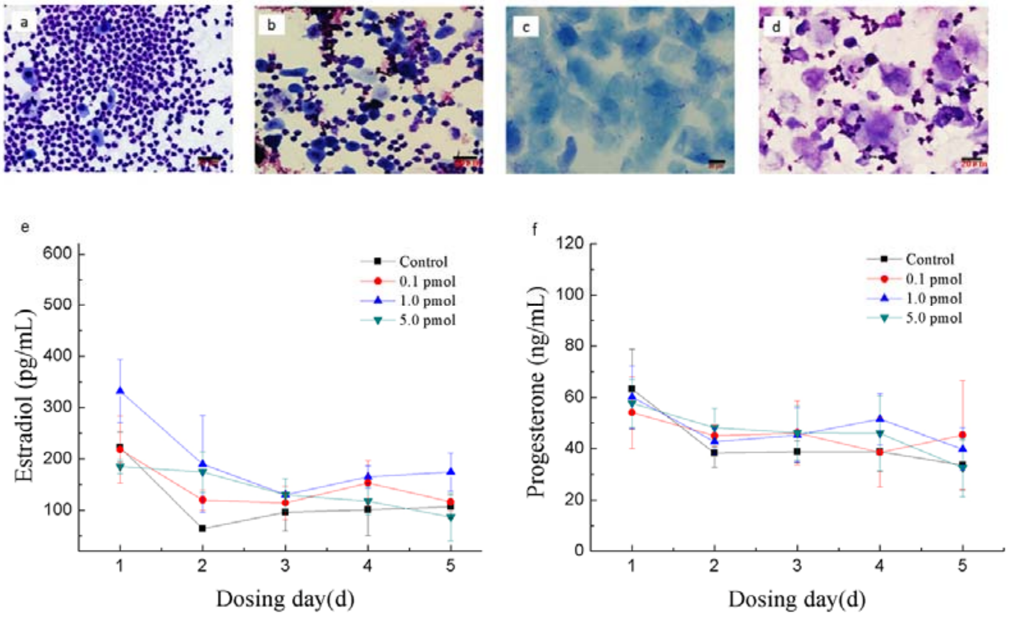

Figure S1. Typical cytology of vaginal secretion at different stage of the estrous cycle for mouse treated with QDs at 5.0 pmol. Scale bar: 20 μm. a) diestrus, b) proestrus, c) estrus, d) metestrus.e) The trend of estradiol after the peak day during treatment. f) The trend of progesterone after the estradiol peak day during treatment. a-d) n=7. e-f) The data was presented as the mean ±SD. n=4.

Methods

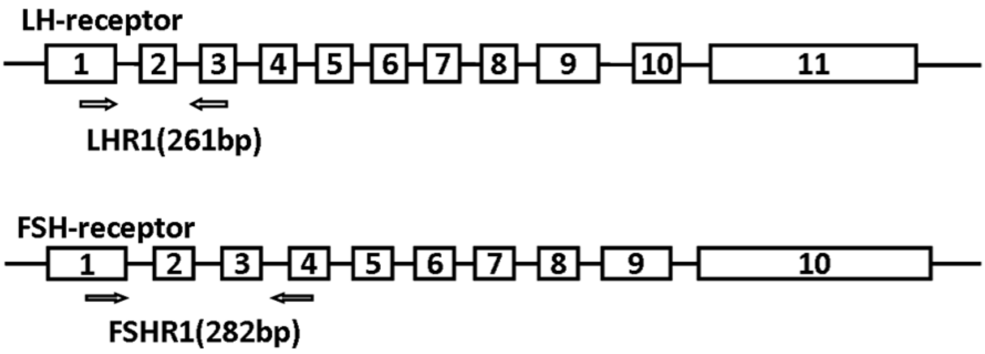

Figure S2. Primer design for real-time PCR.

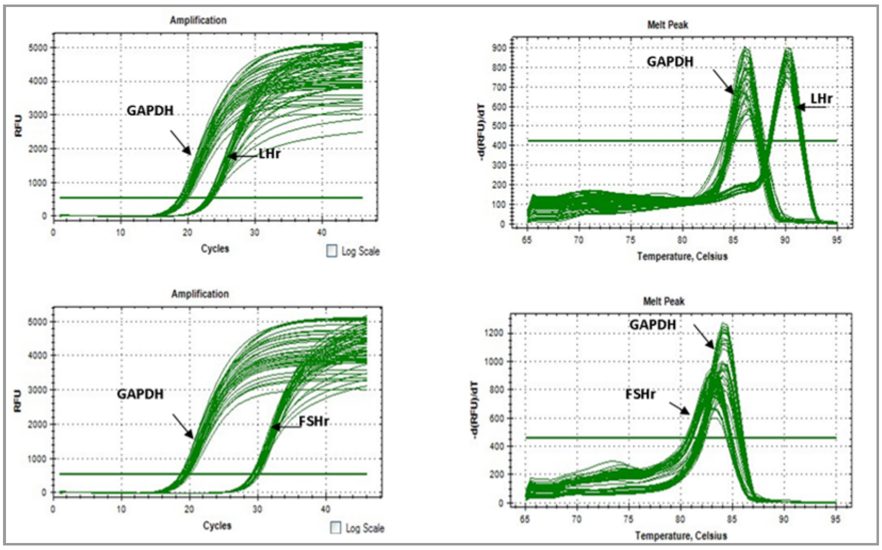

Figure S3. The amplification curve and melting curve of LHr and FSHr.

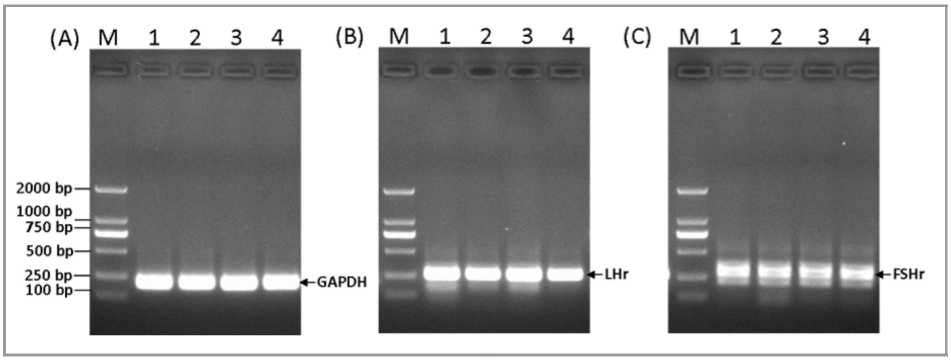

Figure S4. The electrophoretogram of different gene after quantitative PCR.

Lane M: DL2000 maker; lane 1: Control; lane 2, Group of 0.1 nmol L^{-1}; lane 3: Group of 1.0 nmol L^{-1}; lane 4: Group of 5.0 nmol L^{-1}.

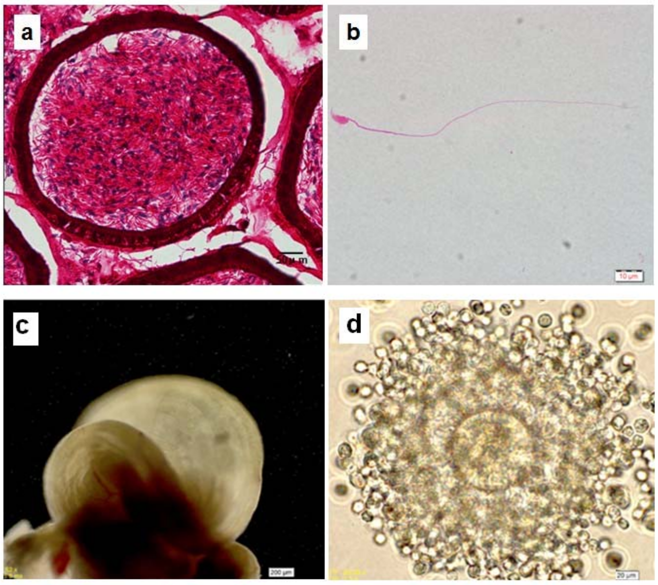

Figure S5. Morphology of epididymis, sperm, oviduct ampulla magnum and COCs. a) Small lumen of the epididymis, Scale bar: 50 μm.; b) sperm, Scale bar: 10 μm.; c) oviduct ampulla magnum, Scale bar: 200 μm.; d) COCs, Scale bar: 20 μm.

Table S1. Amplification reaction volume of RT-PCR

Chemicals	Volume（μL）
cDNA template	1
Sense primer	1
Anti-sense primer	1
SsoFast EvaGreen supermix	10
Rnase Free dH_2O	7
Total volume	20

Table S2. Amplification reaction condition of real-time PCR

Temperature（℃）	Time（s）	Cycles
94	300	1
94	30	45
55	30	
72	45	
72	60	1
